# Early extubation after thymectomy is good for the patients with myasthenia gravis

**DOI:** 10.1007/s10072-019-03941-0

**Published:** 2019-06-10

**Authors:** Li Chen, Wenfeng Xie, Donghua Zheng, Siqi Wang, Ganping Wang, Jiaqi Sun, Qiang Tai, Zhenguang Chen

**Affiliations:** 1 Intensive Care Unit of East Division, The First Affiliated Hospital, Sun Yat-sen University, No. 58, Zhongshan Road II, Guangzhou, 510080 Guangdong China; 2 Department of Thoracic Surgery, The First Affiliated Hospital, Sun Yat-sen University, No. 58, Zhongshan Road II, Guangzhou, 510080 Guangdong China; 3 Department of Cardiothoracic Surgery of East Division, The First Affiliated Hospital, Sun Yat-sen University, Guangzhou, 510080 Guangdong China

**Keywords:** Myasthenia gravis, Extubation, Thymectomy

## Abstract

**Objectives:**

Patients with myasthenia gravis (MG) often benefit from thymectomy, but the optimal timing of extubation following thymectomy in these patients remains unknown. This study of MG patients compared the effect of early and late extubation following thymectomy on clinical outcome.

**Methods:**

We performed a study of data from 96 patients with MG who received thymectomy procedures, followed by early (< 6 h) or late (> 6 h) extubation, at our institution between October 2011 and November 2017. Patient clinical and demographic characteristics, preoperative data, and postoperative clinical outcomes were analyzed. Data sharing is not applicable to this article as no datasets were generated or analyzed during the current study.

**Results:**

The patients in the early extubation group (*n* = 53) and late extubation group (*n* = 43) had similar preoperative clinical and demographic characteristics. However, the early extubation group had a significantly longer duration of MG (24 months vs. 12 months, *P* < 0.013) and a lower incidence of reintubation (11.3% vs. 37.2%, *P* = 0.003). Postoperative pulmonary infection was significantly more common in the late extubation group (39.5% vs. 11.3%, *P* = 0.001; adjusted odds ratio = 6.94, 95% CI 1.24–38.97). Also, patients in the late extubation group had a longer duration of ICU stay (6.4 ± 4.0 h vs. 4.3 ± 1.8 h; *P* = 0.003) and had a longer adjusted duration of ICU stay by 0.93 days (95% CI 0.02–1.85).

**Conclusions:**

Our analysis of patients with MG who received thymectomy procedures indicated that early extubation was associated with improved clinical outcomes, in particular with reduced risk of postoperative pulmonary infection and reduced ICU stay.

**Electronic supplementary material:**

The online version of this article (10.1007/s10072-019-03941-0) contains supplementary material, which is available to authorized users.

## Introduction

Myasthenia gravis (MG) is an autoimmune disorder characterized by varying degrees of weakness and fatigability of skeletal muscles due to the presence of autoantibodies directed against different components of the neuromuscular junction (NMJ) [1, 2]. About 10 to 20% of MG patients suffer from thymoma, and at least 60% of patients present with thymus hyperplasia or dysplasia [3]. Patients with thymomatous or nonthymomatous MG benefit from thymectomy. This procedure modifies the natural course of MG by eliminating receptor sites for acetylcholine, and thereby helps to reduce the autoimmune response [4].

Because of the increased use of surgery and medical costs, Verrier et al. initially promoted the use of “fast-track surgery” in 1993 [5]. The core of fast-track surgery is early extubation, in an effort to reduce the use of a medical ventilator and nursing staff. Overall, fast-track surgery is expected to shorten ICU stays and provide earlier hospital discharge.

Many researchers have confirmed that early extubation reduces the risk for adverse events associated with prolonged intubation, such as laryngotracheal injuries, mucous congestion of the endotracheal tube, ventilator-acquired pneumonia, pulmonary atelectasis, and pulmonary hypertensive crises due to suctioning of the endotracheal tube [6, 7]. Early extubation following surgery can also reduce the need for sedation, lower the risk of delirium, allow early initiation of feeding and parental interactions, and reduce the ICU stay and psychological trauma.

General anesthesia by tracheal intubation is the main method of anesthesia for thymectomy. MG patients have unpredictable responses to muscle relaxants and increased susceptibility to postoperative respiratory failure, which could lead to prolonged dependence on mechanical ventilation [8]. Thus, the optimal timing for extubation following thymectomy of these patients remains unknown. The present study of patients with MG compared the effect of early and late extubation following thymectomy on clinical outcome to provide reliable evidence to assist physicians in making clinical decisions.

## Patients and methods

### Patients and study design

This study was a retrospective analysis of a prospectively maintained database for a surgical ICU with 9 beds in a single tertiary teaching hospital between October 2011 and November 2017. A total of 96 patients were enrolled. The study protocol was approved by the ethics committee of the First Affiliated Hospital of Sun Yat-sen University, which waived the need for informed consent. The trial was registered with ClinicalTrials.gov (No. NCT03468452).

We collected data on all patients who were 18 years or older, admitted with a main diagnosis of MG, and underwent thymectomy. The preoperative information collected from the patient records included demographic data, duration of MG, severity of MG (based on Osserman’s classification [9]), pathological type of MG, history of myasthenic crisis, dose of pyridostigmine, use of a steroid hormone, and use of an immunosuppressant. Although the classification of MG by the Myasthenia Gravis Foundation of America is widely accepted [9], the Osserman and Genkins classification were used because of our institutional guidelines.

The diagnosis of MG was confirmed by clinical presentation and electrodiagnostic tests, such as repeated low- and high-frequency nerve stimulation and electromyograph and simple-fiber electromyography (if necessary). A postoperative myasthenic crisis (POMC) was defined by the present research [10]. Disease duration was defined as the time from the onset of initial symptoms to hospital admission for surgery. The presence of myasthenic crisis before the surgery, which is characterized by increasing muscle weakness and respiratory failure requiring tracheal intubation and mechanical ventilation, was recorded. If the case required tracheal intubation because of myasthenic crisis, the surgical treatment was performed once myasthenia crisis was overcome, rather than during mechanical ventilation and myasthenia crisis. All cases were performed with extended transsternal thymectomy to complete surgical dissection. Pathological data were used to classify the thymus as a thymoma or as thymic hyperplasia. An ectopic thymus was defined by the presence of thymus tissue in the perithymic fat, based on microscopy [11]. The preoperative regimen of anticholinesterase and steroid hormone therapy was optimized, and maintained until the time of the operation. The steroid hormone dose was gradually tapered to the minimal dose required to maintain remission. An immunosuppressant was only used preoperatively in a few patients with severe MG whom steroid therapy was insufficient or ineffective.

The decision regarding the timing of tracheal extubation was based on clinical and respiratory variables. In particular, this procedure considered the presence of clear consciousness, stable spontaneous ventilation, normal gas exchange with low-flow oxygen inhalation, and hemodynamic stability. Arterial blood gas analysis was conducted 2 h after extubation.

### Primary and secondary outcomes

The primary outcome measures are indicators of treatment efficacy: postoperative pulmonary infection, reintubation, total duration of hospital stay, duration of ICU stay, and duration of postoperative hospital stay. The secondary outcome measures are indicators of patient status: POMC, postoperative dose of pyridostigmine, postoperative use of a steroid hormone, postoperative use of intravenous immunoglobulin, and duration of postoperative intravenous immunoglobulin.

### Statistical analysis

The patients were divided into an early extubation group (< 6 h) and late extubation group (> 6 h), as in previous reports [12, 13]. Categorical variables are provided as proportions (%). Continuous variables are provided as medians (interquartile ranges [IQRs]) if the distribution was nonnormal, and as means (SDs) if the distribution was normal. Categorical variables were compared using the chi-square test or Fisher’s exact test. Student’s *t* test and the Wilcoxon rank sum test were used for comparisons of normally and nonnormally distributed continuous variables, respectively.

A nonconditional logistic regression model was generated to assess the relationship of the time of extubation with the rate of postoperative pulmonary infection. Odds ratios (ORs) and 95% confidence intervals (CIs) were calculated, using the early extubation group as the reference.

Linear regression models were used to determine the association between extubation time and the duration of the ICU stay. This analysis estimated the regression coefficient and 95% CI for the late extubation group relative to the early extubation group.

For the nonconditional logistic regression and linear regression analyses, a crude model (univariate), an age- and sex-adjusted model, and a multivariate-adjusted model (with adjustment for variables selected based on clinical relevance and the descriptive univariate analysis) were used to calculate ORs and adjusted ORs (aORs).

All *P* values are two-tailed, and a *P* value below 0.05 was considered statistically significant. Statistical analyses were performed using SPSS v.22.0 (SPSS Inc., Chicago, IL, USA).

## Results

From October 2011 to November 2017, 96 MG patients underwent thymectomy in our institution and met the Osserman’s classification (Table 1). There were 61 females and 35 males, the median age was 30 years (IQR = 22 to 45), and the average weight was 55.8 ± 12.3 kg. The median duration of disease was 24 months, and most patients (58, 58.3%) had Osserman’s class II MG. Analysis of other preoperative variables indicated that 7.3% of patients had thymoma (based on pathologic diagnosis), 23% had a myasthenic crisis requiring mechanical ventilation, 35.4% used an adjunctive steroid hormone therapy, and 8.3% used an immunosuppressant therapy. The mean dose of preoperative pyridostigmine (used by nearly half of the patients) was 180 mg/day.

**Table 1:** Demographic and clinical characteristics of patients

Variables	Patients (*n* = 96)
Male sex, no. (%)	35 (32.4)
Age, median (IQR), years	30 (22–45)
Weight, mean (SD), kg	55.8 (12.3)
Duration of the disease, median (IQR), months	24 (8.25–48)
Osserman’s class, no. (%)	
I	6 (6.3)
II	56 (58.3)
III	29 (30.2)
IV	5 (5.2)
Pathological types, no. (%)	
Thymoma	7 (7.3)
Thymic hyperplasia	89 (92.7)
Preoperative myasthenic crisis, no. (%)	22 (23.0)
Preoperative dose of pyridostigmine, median (IQR), mg	180 (180–240)
Preoperative steroid hormone, no. (%)	34 (35.4)
Preoperative immunosuppressant, no. (%)	8 (8.3)
Operative anesthesia schedule, no. (%)	
≤ 1 h	58 (60.4)
> 1 h	38 (39.6)
PH after extubation, mean (SD)	7.38 (0.05)
PO_2_ after extubation, mean (SD), mmHg	157.7 (57.8)
PCO_2_ after extubation, mean (SD), mmHg	43.3 (4.7)
Postoperative dose of pyridostigmine, median (IQR), mg	180 (180–240)
Postoperative steroid hormone, no. (%)	31 (32.3)
Postoperative intravenous immunoglobulin, no. (%)	69 (71.9)
Duration of postoperative intravenous immunoglobulin, median (IQR), days	3.0 (0–4.75)
Postoperative pulmonary infection, no. (%)	23 (24)
Reintubation, no. (%)	22 (22.9)
POMC no. (%)	9 (9.4)
Length of hospital stay, mean (SD), days	22 (12)
Length of ICU stay, mean (SD), days	5.2 (3.1)
Length of Postoperative hospital stay, mean (SD), days	13.1 (7.0)

*ICU* intensive care unit, *POMC* postoperative myasthenic crisis

At 2 h after extubation, all measured parameters (pH, PO_2_, and PCO_2_) from arterial blood gas analysis were normal in all patients. In addition, the preoperative and postoperative doses of pyridostigmine were very similar. Analysis of other postoperative variables indicated that 32.3% of patients used a steroid hormone therapy, 71.9% used intravenous immunoglobulin therapy, 24% had pulmonary infections, 22.9% required reintubation for respiratory muscle weakness or other conditions, and 9.4% had POMC. Overall, the average total hospital stay was 22 ± 12 h, the average ICU stay was 5.2 ± 3.1 h, and the average postoperative stay was 13.1 ± 7.0 h.

There were 53 patients in the early extubation group and 43 patients in the late extubation group (Table 2). Analysis of the demographic and preoperative characteristics of these groups indicated they were similar with regard to age, sex, weight, severity of the disease (based on Osserman’s classification), pathological types of MG, history of preoperative myasthenic crisis, preoperative dose of pyridostigmine, preoperative use of a steroid hormone, and preoperative use of an immunosuppressant. However, disease duration was significantly longer in the early extubation group (*P* < 0.013).

**Table 2:** Demographics, preoperative, and operative characteristics of early extubation group and late extubation group

Variables	Early extubation (*n* = 53)	Late extubation (*n* = 43)	*P* value
Male sex, no. (%)	19 (15.8)	16 (15.7)	0.896
Age, median (IQR), years	29 (19.5–43.5)	32 (25–47)	0.196
Weight, mean (SD), kg	55.8 (13.6)	55.7 (11.4)	0.947
Duration of the disease, median (IQR), months	24 (12–54)	12 (6–48)	0.013
Osserman’s classification, no. (%)			
I	4 (7.5)	2 (4.7)	0.575
II	31 (58.4)	25 (58.1)	
III	16 (30.2)	13 (30.2)	
IV	2 (3.8)	3 (7.0)	
Pathological types, no. (%)			
Thymoma	3 (5.7)	4 (9.3)	0.774
Thymic hyperplasia	50 (94.3)	39 (90.7)	
Operative anesthesia schedule			
≤ 1 h	31 (58.5)	27 (62.8)	0.668
> 1 h	22 (41.5)	16 (37.2)	
Preoperative myasthenic crisis, no. (%)	9 (17)	13 (30.2)	0.124
Preoperative dose of pyridostigmine, median (IQR), mg	180 (180–240)	240 (180–240)	0.010
Preoperative steroid hormone, no. (%)	18 (34)	16 (37.2)	0.741
Preoperative immunosuppressant, no. (%)	4 (7.5)	4 (9.3)	0.758

Analysis of the postoperative parameters of the two groups indicated no significant differences in arterial blood gas data (pH, PO_2_, and PCO_2_), dose of pyridostigmine, use of a steroid hormone, use of intravenous immunoglobulin, duration of intravenous immunoglobulin, incidence of POMC, duration of hospital stay, and duration of postoperative hospital stay (Table 3). However, fewer patients in the early extubation group required reintubation (11.3% vs. 37.2%, *P* = 0.003) and had postoperative pulmonary infections (11.3% vs. 39.5%; *P* = 0.001). In addition, the average ICU stay was shorter in the early extubation group (6.4 ± 4.0 h vs. 4.3 ± 1.8 h, *P* = 0.003).

**Table 3:** Postoperative parameters of early extubation group and late extubation group

Variables	Early extubation (*n* = 53)	Late extubation (*n* = 43)	*P* value
PH after extubation, mean (SD)	7.36 (0.04)	7.40 (0.05)	0.947
PO_2_ after extubation, mean (SD), mmHg	158.5 (62.3)	156.7 (52.4)	0.881
PCO_2_ after extubation, mean (SD), mmHg	43.8 (4.9)	42.7 (4.7)	0.277
Postoperative dose of pyridostigmine, median (IQR), mg	180 (180–240)	210 (180–240)	0.071
Postoperative steroid hormone, no. (%)	16 (30.2)	15 (34.9)	0.625
Postoperative intravenous immunoglobulin, no. (%)	34 (64.2)	35 (81.4)	0.062
Duration of postoperative intravenous immunoglobulin, median (IQR), days	2 (0–5)	3 (2–4)	0.098
Postoperative pulmonary infection, no. (%)	6 (11.3)	17 (39.5)	0.001
Reintubation, no. (%)	6 (11.3)	16 (37.2)	0.003
POMC no. (%)	5 (9.4)	4 (9.3)	0.342
Length of hospital stay, mean (SD), days	21.5 (10.3)	22.7 (14.8)	0.634
Length of ICU stay, mean (SD), days	4.3 (1.8)	6.4 (4.0)	0.003
Length of postoperative hospital stay, mean (SD), days	13.0 (6.0)	13.3 (8.3)	0.834

*POMC* postoperative myasthenic crisis

Further analysis of postoperative data using multiple logistic regression indicated that the late extubation group had a higher rate of postoperative pulmonary infections (*P* = 0.001; aOR = 6.94, 95% CI = 1.24–38.97) (Table 4). Moreover, patients in the late extubation group had a longer duration of ICU stay compared with those in the early extubation group (6.4 ± 4.0 vs. 4.3 ± 1.8; *P* = 0.003) and had a longer adjusted duration of ICU stay by 0.93 days (95% CI = 0.02–1.85) (Table 5).

**Table 4:** Postoperative pulmonary infection according to initiation of extubation after thymectomy

	Early extubation (*n* = 53)	Late extubation (*n* = 43)	*P* value
Postoperative pulmonary infection, no. (%)	6 (11.3)	17 (39.5)	
Crude OR (95% CI)	1.00 (ref.)	5.12 (1.80–14.60)	0.002
Age- and sex-adjusted OR (95% CI)	1.00 (ref.)	5.00 (1.74–14.31)	0.003
Multivariate-adjusted OR (95% CI)	1.00 (ref.)	6.94 (1.24–38.97)	0.028

**Table 5:** Estimates (regression coefficients and 95% CI) for length of ICU stay according to initiation of extubation after thymectomy

	Early extubation (*n* = 53)	Late extubation (*n* = 43)	*P* value
Length of ICU stay, mean (SD), days	4.3 (1.8)	6.4 (4.0)	
Crude estimate (95% CI)	0 (ref.)	+ 2.05 (+ 0.83 to + 3.27)	0.001
Age- and sex-adjusted estimate (95% CI)	0 (ref.)	+ 2.13 (+ 0.90 to + 3.36)	0.001
Multivariate-adjusted estimate (95% CI)	0 (ref.)	+ 0.93 (+ 0.02 to + 1.85)	0.046

## Discussion

The major result of this retrospective clinical trial of MG patients receiving thymectomy is that early extubation following surgery reduced the risk of postoperative pulmonary infection and shortened the duration of the ICU stay.

Myasthenia gravis (MG) is an autoimmune disorder characterized by progressive muscle fatigue due to alterations of the postsynaptic NMJ. Most patients also experience abnormalities of the thymus gland, such as thymoma or benign thymus hyperplasia [14]. Several studies indicate that thymectomy during early-stage MG can improve overall prognosis [15]. The extended transsternal thymectomy is a routine procedure used for complete surgical dissection [16]. A minimally invasive method, such as video-assisted thoracoscopic surgery (VATS), may be used to avoid a sternotomy, and to reduce cosmetic damage, pain [17], blood loss, chest tube duration, and length of hospital stay, while providing clinical outcomes comparable with sternotomy in appropriately selected patients [18]. There is little known about the effect of the timing of extubation following thymectomy in patients with MG.

Traditionally, a patient would receive an elective surgery with endotracheal intubation in the morning, depart the operating room by noon, breathe with machine assistance overnight, and be extubated the following morning [19]. This method is generally recognized to reduce the need for spontaneous breathing, stress, and the workload of the heart [20]. If extubation is performed soon after surgery, it increases the risk of pulmonary complications, the need for mechanical ventilation, and pain, and requires more resources from the ICU staff. However, with improvements in surgery, many studies have demonstrated the advantages, feasibility, and safety of early extubation [21–23]. Thus, most recent studies show that patients benefit when endotracheal intubation is removed as early as possible [20, 22]. Early extubation facilitates recovery of the respiratory tract mucosa and decreases postsurgical pulmonary complications, such as pulmonary atelectasis, pneumonia, and pleural effusion [24, 25]. Moreover, cardiac diastolic compliance is also improved. After surgery, the patient is typically weak and has sore muscles. Thus, patients with tracheal intubations have reduced number of active breaths, due to the need to cooperate with the ventilator, and oral secretions cannot be eliminated. In addition to pain at the incision, a patient may fidget and even experience delirium, and this increases pain and postoperative complications [26]. Early extubation can improve patients’ self-care, accelerate blood circulation in the lungs, and promote more rapid recovery.

Despite the increasing evidence that fast-track surgery, including early extubation, can be achieved safely, there are still individual and institutional concerns about the safety of this approach. Large series in adult cardiac surgery have shown that early extubation can be accomplished safely, and provides significant benefits to the patient [21–23]. There are special concerns regarding thymectomy in patients with MG, because this autoimmune disease is characterized by muscle weakness and fatigability, so patients may require prolonged dependence on mechanical ventilation. Thus, if endotracheal intubation is removed within 6 h after surgery, MG patients could develop postoperative complications, such as acid–base imbalance, respiratory failure, and POMC. However, research by Kas et al. [27] indicated that the high incidence of airway complications, especially ventilator-associated pneumonia (VAP), after thymectomy is mostly due to the long duration of mechanical ventilation. On the one hand, patients with tracheal intubation may have a decreased cough reflex, causing aspiration or poor sputum capacity, so there is no timely removal of airway secretions. On the other hand, long-term mechanical ventilation may lead to the contamination of medical devices, such as ventilator tubes, and deficient airway humidification. VAP itself increases the risk of death, and the resulting airway stenosis may also lead to a myasthenic crisis.

Our single-center, retrospective study of 96 MG patients indicated that the groups receiving early and late extubation were similar with regard to age, sex, body weight, duration of MG, severity of MG, pathological types of MG, history of preoperative myasthenic crisis, preoperative dose of pyridostigmine, preoperative use of a steroid hormone, and preoperative use of an immunosuppressant. However, the incidence of postoperative pulmonary infection was significantly lower in the early extubation group. Moreover, the early extubation group had a shorter ICU stay. Thus, our results are in accordance with previous studies that “fast-tracking” of cardiac surgery patients (i.e., early extubation) provides excellent patient outcomes, reduces the risk of ventilator-associated infections, reduces patient time in the ICU, reduces the need for nursing staff, and more efficiently uses limited hospital resources [28].

A recent study by Jiang et al. [29] examined the use of spontaneous-ventilation video-assisted thoracic thymectomy (SV-VATT) in patients with MG, and demonstrated successful thymectomy without endotracheal intubation. However, this procedure requires excellent anesthetic technique and management, and can be technically challenging for a surgeon. Moreover, this previous study was only a small retrospective cohort study, and this technique is not as widely used as thymectomy. At present, general anesthesia by tracheal intubation is the main method of anesthesia for thymectomy, although the timing of extubation of patients with MG undergoing thymectomy requires further investigation.

The rapid discontinuation of mechanical ventilation, in the OR or soon after ICU arrival, is a core feature of fast-track surgery. Regardless of when the patient is extubated, pain management, sedation without respiratory depression, and mild respiratory acidosis are important considerations. More importantly, the rate of reintubation following early extubation in patients undergoing surgery is typically very low, ranging from 2 to 8% [30]. The somewhat higher incidence of reintubation of patients receiving early extubation in our study (11.3%) may be related to the presence of underlying MG, the severity of MG, or other factors. Therefore, we suggest use of a scoring system that considers demographic factors, preoperative characteristics, intraoperative parameters, and postoperation outcomes to select the optimal time for extubation. The application of such a scoring system in MG patients may prolong mechanical ventilation for appropriate patients, reduce the need for reintubation, and preserve the advantages of early extubation for high-risk patients.

There were several limitations of this study. First, the study was retrospective and examined the records of patients from a single institution. Although the baseline characteristics of the two groups were similar, several sources of bias existed. A multicenter randomized controlled trial (RCT) is needed to verify our results. Second, there are no “gold-standard” criteria for extubation following thymectomy, especially for patients with MG. Thus, the timing of extubation is partly based on the subjective assessment of the attending intensivist. Finally, our use of a 6-h threshold to distinguish early and late extubation was somewhat arbitrary. Larger and prespecified groups of MG patients are required to determine the effect of using other thresholds for comparison of early and late extubation.

## Conclusion

We conclude that early extubation after thymectomy is feasible in patients with MG. Early extubation reduces the risk of postoperative pulmonary infection and the length of stay in the ICU.

## Electronic supplementary material

ESM 1 (XLSX 19 kb)

## Abbreviations

CI: confidence interval
ICU: intensive care unit
MG: myasthenia gravis
NMJ: neuromuscular junction
OR: odds ratio
POMC: postoperative myasthenic crisis
